# Rectal indomethacin versus placebo to reduce the incidence of pancreatitis after endoscopic retrograde cholangiopancreatography: results of a controlled clinical trial

**DOI:** 10.1186/s12876-015-0314-2

**Published:** 2015-07-21

**Authors:** Víctor Fernando Andrade-Dávila, Mariana Chávez-Tostado, Carlos Dávalos-Cobián, Jesús García-Correa, Alejandro Montaño-Loza, Clotilde Fuentes-Orozco, Michel Dassaejv Macías-Amezcua, Jesús García-Rentería, Jorge Rendón-Félix, José Antonio Cortés-Lares, Gabriela Ambriz-González, Ana Olivia Cortés-Flores, Andrea del Socorro Alvarez-Villaseñor, Alejandro González-Ojeda

**Affiliations:** 1 Department Gastrointestinal Endoscopy, Specialties Hospital of the Western National Medical Center, Mexican Institute of Social Security, Guadalajara, Jalisco México; 2 Research Unit in Clinical Epidemiology, Specialties Hospital of the Western National Medical Center, Mexican Institute of Social Security, CP 44340 Guadalajara, Jalisco México; 3 Department of Pediatric Surgery, Children’s Hospital of the Western National Medical Center, Mexican Institute of Social Security, Guadalajara, Jalisco México; 4 Health Research Coordination, Mexican Institute of Social Security, Baja California Sur, Mexico

**Keywords:** Prophylaxis, Rectal indomethacin, Postendoscopic retrograde cholangiopancreatography pancreatitis

## Abstract

**Background:**

Acute pancreatitis is the most common major complication after endoscopic retrograde cholangiopancreatography (ERCP). Many drugs have been evaluated for prophylaxis, including nonsteroidal anti-inflammatory drugs (NSAIDs), which are potent inhibitors of phospholipase A_2_ and play a role in the pathogenesis of acute pancreatitis. Rectal NSAIDs have been shown in prospective studies to decrease the incidence of this complication, but the indication is not generalized in clinical practice. The aim of this study was to evaluate the efficacy of rectal administration of indomethacin in reducing the incidence of post-ERCP pancreatitis in high-risk patients.

**Methods:**

This was a controlled clinical trial where patients with an elevated risk of developing post-ERCP pancreatitis were assigned to receive 100 mg of rectal indomethacin or a 2.6 g suppository of glycerin immediately after ERCP, without placement of a pancreatic stent. The patients were determined to be at high risk based on validated patient- and procedure-related risk factors. Post-ERCP pancreatitis was defined as the presence of new upper abdominal pain, hyperamylasemia/hyperlipasemia (at least three times the upper limit) 2 hours after the procedure and hospitalization at least 48 hours because of the complication. Pancreatitis severity was defined according to Cotton’s criteria.

**Results:**

One hundred sixty-six patients were included; 82 in the study group and 84 in the placebo group. Patients had at least one major and/or two minor risk factors for developing post-ERCP pancreatitis. The incidence of the complication was 4.87 % (4/82) in the study group and 20.23 % (17/84) in the placebo group; this difference was significant (*P* = 0.01). According to Cotton’s criteria, 17 patients (80.9 %) developed mild pancreatitis and 4 (19.1 %) had moderate pancreatitis; 3 of these 4 patients belonged to the placebo group (*P* = 0.60). Based on these results, an absolute risk reduction of 0.15 (15 %), a relative risk reduction of 0.75 (75 %) and a number needed to treat of 6.5 patients were calculated to prevent an episode of post-ERCP pancreatitis. There was no mortality.

**Conclusions:**

Rectal indomethacin reduced the incidence of post-ERCP pancreatitis among patients at high risk of developing this complication.

**Trial registration:**

National Clinical Trials NCT02110810. Date April 7, 2014.

## Background

Endoscopic retrograde cholangiopancreatography (ERCP) is now widely accepted as a therapeutic modality of benign and malignant diseases of the pancreatobiliary tree. Acute pancreatitis represents the most common and feared complication following ERCP. The reported incidence of this complication is 1–40 % according to the presence of high-risk factors or the presence of sphincter of Oddi dysfunction (SOD) [1]. In most of the prospective series, the incidence reported ranged between 3.5 % and 20 % for nonselected and for high-risk patients, respectively. Independent risk factors for post-ERCP pancreatitis (PEP) are either patient- or procedure-related [1, 2].

Although most episodes of PEP are mild (80–90 %), a small proportion of patients develop severe pancreatitis, resulting in prolonged hospitalization, a long stay in the Intensive Care Unit and utilization of major hospital resources. These patients have increased morbidity and mortality rates [3]. Despite technical improvements and increased skills for endoscopists, the incidence of PEP has not yet decreased substantially [4].

To date, pancreatic stent placement appears to be the best way to reduce the incidence of this complication in high-risk patients; it is currently recommended by some guidelines [5, 6] and is considered the standard of care for the prevention of PEP [7].

However, pancreatic stenting is a difficult maneuver to perform and comes relatively late (at the end of the endoscopic procedure), mainly in patients with difficult cannulation of the biliary–pancreatic ducts; in addition, many endoscopists are not familiar with this procedure. This unique maneuver may leave the patient worse off than if no attempt was made to perform the procedure [8].

There is no gold standard to prevent this complication. For this reason, more than 35 pharmacologic agents have been studied in many prospective clinical trials. To date, no medication has proven to be consistently effective in preventing PEP and no pharmacological prophylaxis is in widespread clinical use [9–12].

Nonsteroidal anti-inflammatory drugs (NSAIDs) are potent inhibitors of phospholipase A_2_, cyclooxygenase and neutrophil–endothelial interactions, all believed to play an important role in the pathogenesis of acute pancreatitis. NSAIDs are inexpensive and easily administered and have a favorable risk profile when given as a single dose, making them an attractive option in the prevention of PEP. Preliminary studies evaluating the protective effects of single-dose rectal indomethacin or diclofenac in PEP have been conducted, and meta-analyses suggest a benefit [12, 13].

Prophylaxis of PEP constitutes a continuous challenge. The ideal prophylactic agent should be a drug with a low cost, be easily administrated and with mild or no adverse effects. The identification of patients at a high risk of this complication is difficult before the endoscopic procedure because many risk factors are procedure-related. There is some evidence to suggest a beneficial effect from the use of NSAIDs applied rectally immediately after the conclusion of ERCP [14–16].

The aim of this study was to evaluate the efficacy of rectally administered indomethacin in reducing the incidence of PEP in high-risk patients.

## Methods

### Design

This was a controlled clinical trial conducted between July 2012 and December 2013 in patients scheduled for ERCP at the Department of Gastrointestinal Endoscopy and Department of Gastroenterology of the Specialties Hospital of the Western Medical Center in Guadalajara, Jalisco, Mexico.

The inclusion criteria selected patients with an elevated baseline risk of PEP on the basis of prospectively validated patient- and procedure-related independent risk factors [17, 18].

Patients were eligible if they met one or more of the following criteria: clinical suspicion of SOD, a history of PEP, pancreatic sphincterotomy, precut sphincterectomy, more than eight cannulation attempts, pneumatic dilatation of an intact biliary sphincter, or ampullectomy. In addition, they were also eligible for inclusion if they met at least two of the following criteria: age less than 50 years and female sex, a history of recurrent pancreatitis (> two episodes), three or more injections of contrast agent into the pancreatic duct with at least one injection to the tail of the pancreas, excessive injection of a contrast agent into the pancreatic duct resulting in opacification of pancreatic acini, or the acquisition of a cytologic specimen from the pancreatic duct with the use of a brush.

The exclusion criteria were: unwillingness or inability to consent to the study, age less than 18 years, pregnancy, breastfeeding mother, standard contraindication for ERCP, hypersensitivity to aspirin or NSAIDs, previous use of NSAIDs within 1 week, creatinine level ≥ 1.6 mg/dl, active or recent (4 weeks) gastrointestinal hemorrhage, chronic calcified pancreatitis, pancreatic head malignancy, procedure performed on major papilla/ventral pancreatic duct in a patient with pancreas divisum, ERCP for biliary stent removal or exchange without anticipated pancreatogram, subjects with prior biliary sphincterotomy now scheduled for repeat biliary therapy without anticipated pancreatogram and anticipated inability to follow the study protocol.

The ERCP procedures were performed with the patient under topical pharyngeal anesthesia with 2 % lidocaine and after administration of sedation (midazolam) and analgesia (fentanyl) intravenously, with dosage at the discretion of the endoscopist. Patients received complementary oxygen (3 to 5 l/min) through a nasal external device and infusion of 200 to 500 ml of 0.9 % saline solution. The material used to perform ERCP consisted of a video duodenoscope model TJF-Q180V (Olympus™), traction sphincterotome for selective cannulation of the bile duct, needle scalpel to perform the precut sphincterotomy, hydrophilic guide wire via the bile duct, Dormia basket and/or balloon catheter for stone extraction, and nonionic water-soluble contrast in concentration of 300 mg I/ml (Optiray™ 300) for opacification of the biliary and pancreatic ducts. Pancreatic stents were only used to treat pancreatic fistulas, not to prevent any pancreatitis events in any cases. All patients were monitored continuously during the procedure, with measurements of blood pressure, heart rate, respiratory rate and arterial oxygen saturation.

Eligible patients provided written informed consent before ERCP and underwent randomization at the conclusion of the endoscopic procedure. Patients without risk factors were not included in the study, based on procedure-related factors alone. If patients met the inclusion criteria, they were randomly assigned to receive a 100 mg suppository of indomethacin or a 2.4 g glycerin suppository of identical appearance after ERCP while patients were under the effect of sedative medication. All patients were kept under surveillance until they became completely awake to prevent spontaneous expulsion of the suppository. The patients, staff endoscopists, residents and researchers were blinded to the treatment assigned to each participant.

The following information was collected. 1) Clinical history, particularly patient-related risk factors for PEP, blood exams for determination of basal amylase, liver enzymes and bilirubin levels, as well as results of ultrasound examination of the liver and biliary tract. 2) All information generated during the ERCP was recorded, particularly parameters related to the procedure risk factors for developing acute pancreatitis. In addition, other nonpancreatic complications were recorded, such as perforation and bleeding. 3) Adverse events related to the rectally applied indomethacin or glycerin suppositories were recorded, such as expulsion, irritation and bleeding.

PEP was considered the main outcome variable and was defined as the development of new or increased abdominal pain consistent with pancreatitis, and elevated amylase or lipase greater than three times the normal upper limit until 24 hours after the procedure, and hospitalization (or prolongation of existing hospitalization) for at least 2 nights. The severity was determined according to consensus guidelines, with mild PEP resulting in a hospitalization of < 3 days, moderate PEP resulting in a hospitalization of 4–10 days, and severe PEP resulting in a hospitalization of > 10 days or leading to the development of pancreatic necrosis or pseudocyst, or requiring percutaneous or surgical intervention. Patients who presented acute pancreatitis after CPRE procedure, were followed up for 30 days after hospital discharge. Asymptomatic hyperamylasemia was defined as any amylase level at least three times above the normal serum level in the absence of abdominal pain, as defined by the consensus criteria [19].

### Follow-up

Patients were kept under surveillance in the endoscopy recovery area for 3 hours after ERCP. Measurement of serum amylase was performed at 2 hours after ERCP in all study patients. Outpatients who were asymptomatic after 4 to 6 hours of surveillance were discharged to home with monitoring for signs and symptoms of acute PEP by phone for 24 hours. Hospitalized patients who were asymptomatic after 4 to 6 hours of surveillance remained in their assigned bed where clinical surveillance was continued for up to 24 hours. If new abdominal pain suggestive of pancreatic origin appeared at any moment during the surveillance period, the 2-hour amylase level was noted and confirmed with serum lipase determination in the following hours. In addition, all usual laboratory exams were performed when acute pancreatitis of any etiology was established. All patients diagnosed with PEP were managed under the medical care of the Department of Gastroenterology.

### Sample size

We evaluated if the application of rectal indomethacin had better results than placebo for the prophylaxis of PEP, with a known incidence of PEP of 25 % in high-risk patients and about 7.5 % in the study group [1, 2]. By calculating the sample size for the comparison of proportions with desired errors α of 0.05 and β of 0.20, a minimum sample size of 80 patients per group was obtained.

### Statistical analysis

The descriptive phase of the analysis included the presentations of data as raw values, percentages and mean ± standard deviation. In the inference phase, Student’s *t* test was used for continuous variables, and χ^2^ or Fisher’s exact tests were used for qualitative variables when appropriate. Furthermore, the absolute risk reduction (ARR), relative risk reduction (RRR) and number needed to treat (NNT) were calculated. Results were considered significant when *P* < 0.05. Statistical analysis was conducted using Excel® 2007 (Microsoft®, Redmond, WA, USA) and SPSS® version 17 for Windows (SPSS Inc., Chicago, IL, USA).

### Ethical considerations

The local Ethics Committee approved the study protocol (identification number 2010-1301-14). All patients gave written informed consent and were randomized using the technique of random numbers in sealed envelopes. The project was carried out with the financial resources of each department and unit, and the authors declare no conflict of financial interest. In addition, the protocol was registered at ClinicalTrials.gov (Identifier NCT02110810).

## Results

**Fig. 1:**
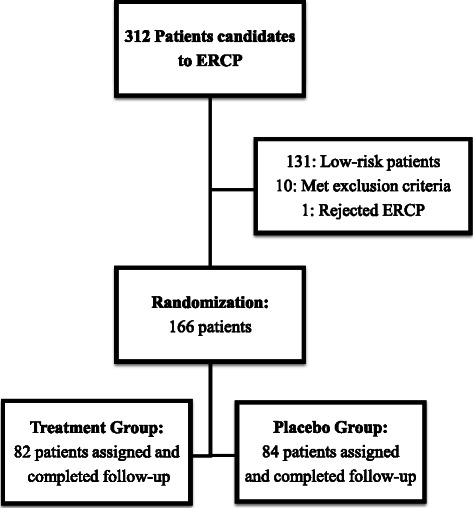
Distribution of patients included in the study

During the study period, 166 consecutive patients who met the inclusion and exclusion criteria were included, as shown in Fig. 1. Eighty-two patients (49.4 %) received 100 mg indomethacin rectally (study group), and 84 patients (50.6 %) received a 2.6 g glycerin suppository (control group). The complete cohort consisted of 110 females (66.2 %) and 56 males (33.8 %). There were 51 females and 31 males (62.1 % and 37.8 %, respectively) in the study group and 59 females and 25 males (70.2 % and 29.7 %, respectively) in the control group. The mean age of patients was 51.6 ± 18.5 years in the study group and 54.0 ± 17.8 years in the control group. The most frequent diagnosis was choledocholithiasis, observed in 34 cases (41.46 %) in the study group and 32 patients (38.1 %) in the control group, followed by benign biliary tract stenosis, suspected SOD and malignant stenosis of the biliary tract. Table 1 summarizes the baseline characteristics of both groups. No significant differences were found when variables were compared.

**Table 1:** Baseline characteristics of patients in the treatment and control groups

Characteristics	Indomethacin group (N = 82)	Glycerin group (N = 84)	*P*
**Female** **Male**	51 (62.19 %) 31 (37.80 %)	59 (70.23 %) 25 (29.76 %)	0.273
**Age (years)**	51.59 ± 18.55	54.0 ± 17.85	0.394
**Outpatients**	46	45	0.74
**Hospitalized**	36	39	
**Without comorbidity**	56	54	0.427
**Comorbid conditions**	26	30	
Diabetes Mellitus type 2	12	14	
Hypertension	7	7	
Dyslipidemia	2	2	
Hypothyroidism	1	0	
COPD	1	1	
Hepatic cirrhosis^a^	1	2	
Ischemic heart disease	1	1	
HIV	0	1	
Asthma	1	2	
**Normal total bilirubin pre-ERCP**	26	24	0.660
**Elevated total bilirubin pre-ERCP**	56	60	
**Previous cholecystectomy**	42	40	0.643
**Dilated bile duct by imaging studies pre-ERCP**	56	62	0.506
**Post-ERCP diagnostics**			0.35
Choledocolithiasis	34	32	
Begin biliary stenosis and/or leakage	18	14	
Suspected sphincter of Oddi dysfunction	12	15	
Normal cholangiogram and/or pancreatogram	8	11	
Malignant biliary tract stenosis	8	9	
Pancreatic fistula	2	3	
**Pre-ERCP amylase level (U/L)**	57.39 ± 21.56	55.36 ± 20.77	0.540

^a^Etiology in the indomethacin group: chronic hepatitis due to hepatitis C virus in 1 patient. Glycerin group etiologies in chronic hepatitis due to hepatitis C virus in 1 patient and primary biliary cirrhosis in 1 patient.

**Fig. 2:**
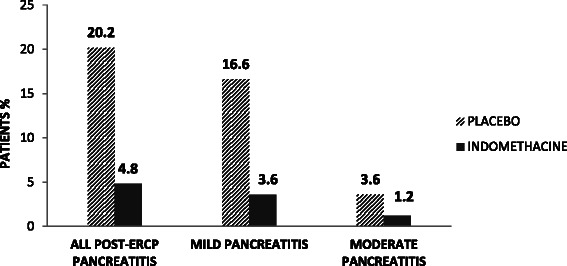
Incidence and distribution of mild and moderate PEP between groups

Twenty-one patients developed PEP, 4 in the treatment group (4.87 %) and 17 in the control group (20.23 %); this difference was significant (*P* = 0.01). Seventeen (80.9 %) cases of pancreatitis occurred in females and 4 cases (20.2 %) in males (*P* = 0.14). Based on these results, an ARR of 0.15 (15 %), an RRR of 0.75 (75 %) and an NNT of 6.5 patients were calculated to prevent an episode of PEP. According to Cotton’s classification, the PEP was mild in 17 patients (80.9 %) and moderate in 4 patients (19.1 %); of these, there were 3 cases in the control group and 1 case in the treatment group (*P* = 0.60), as shown in Fig. 2.

The mean age of the 21 patients with PEP was 48.3 ± 16.2 years, lower than the mean age of 53.6 ± 18.4 years for the 145 patients without pancreatitis; however, the difference was not significant (*P* = 0.21).

The length of hospital stay for those patients who suffered mild pancreatitis was 2.7 ± 0.95 days and 3.8 ± 1.3 days for moderate pancreatitis (*P* = 0.14). There was no mortality as a result of PEP.

Table 2 shows the diagnoses of the patients with pancreatitis; these were distributed similarly between the groups (*P* = 0.35). At 2 hours after ERCP, the mean serum amylase was 141.9 ± 92.6 U/l in the study group and 216.5 ± 105.2 U/l in the control group (*P* < 0.001). In patients who developed pancreatitis, the mean serum amylase at 2 hours after ERCP was 1187.6 ± 789.3 U/l and a mean serum lipase level of 5052.6 ± 2805.1 U/l was measured in the first 24 hours after ERCP. Asymptomatic hyperamylasemia occurred in 100 patients (60.6 %), corresponding to 19 patients in the study group and 81 in the control group (*P* < 0.001).

**Table 2:** Post-ERCP diagnostics of patients in the study and control groups

	Treatment group (N = 4)	Control group (N = 17)	*P value*
Choledocholithiasis	2	7	0.35
Benign biliary stenosis and/or leakage	1	4	
Suspected sphincter of Oddi dysfunction	0	5	
Normal cholangiogram and/or pancreatogram	1	1	
**Severity of the episodes of Pancreatitis**			
Mild	3	14	0.60
Moderate	1	3	

Risk factors for PEP are described in Table 3. They were distributed similarly in both groups with no significant differences. In addition, no differences in the distribution of sex or age were observed. No significant differences were observed when analyzing the development of complications in patients older and younger than 50 years (*P* = 0.44). However, the patients who developed pancreatitis were younger (48.3 ± 16.2 versus 53.7 ± 18.3; *P* = 0.21). There was no difference in the distribution of inpatients and outpatients (*P* = 0.51) or a history of previous cholecystectomy (*P* = 0.12). However, we observed significant differences in several outcome results such as the number of attempts to cannulate the bile duct, in the performance of precut sphincterotomy, the time to cannulate the bile duct and the total duration of the procedure (*P* = 0.001), as well as if patients required pancreatography, also in the number of attempts to pass guide wires and in the injection of contrast material into the pancreatic duct. There was no difference in the extension of pancreatography (*P* = 0.39). Two patients in each group required pancreatic stenting because pancreatic fistulas were diagnosed during ERCP (*P* = 0.62).

**Table 3:** Patient- and procedure-related risk factors identified for the development of PEP

Risk factor	Study Group (N = 82)	Control Group (N = 84)	*P* value
**Patient-related**			
Oddi - Female sex	51	59	0.27
Oddi - Suspected sphincter dysfunction oddi	12	15	0.72
- History of recurrent acute Pancreatitis	4	5	0.76
- Previous post-ERCP pancreatitis	2	1	0.54
- Normal serum bilirubin.	26	24	0.66
**Procedure-related**			
- Attempts to cannulation	7.3 ± 3.6	7.2 ± 3.5	0.97
- Time cannulation	6.0 ± 3.5	6.8 ± 3.6	0.14
- Difficult cannulation of the bile duct (>8 attempts)	38	40	0.86
- Failed cannulation of the bile duct	4	4	0.58
- Precut (access) sphincterotomy	49	46	0.51
- Biliary sphincterotomy	49	48	0.73
- Diameter of the bile duct	11.5 ± 5.3	11.6 ± 4.2	0.84
- Biliary Stent	26	22	0.08
- Pancreatography	41	38	0.34
- Number of passes	1.4 ± 0.5	1.5 ± 0.8	0.25
- Number of injections	1.5 ± 0.8	1.6 ± 0.5	0.52
- Pancreatography extension			
* Partial	8	6	0.14
* Full	31	30	
* Acinarizacion	2	2	
- Pancreatic sphincterotomy	7	5	0.36
- Brushed wirsung duct	8	9	0.33
- Pancreatic stenting	2	2	0.62
- Total procedure time	23.2 ± 6.7	24.6 ± 7.3	0.22

**Table 4:** Comparison between groups with and without PEP

Characteristics	Patients with post-ERCP pancreatitis (N = 21 )	Patients without post-ERCP pancreatitis (N = 145)	*P*
Female	17	93	0.14
Male	4	52	
Age (years)	48.3 ± 16.2	53.7 ± 18.3	0.21
<50 years	11	63	0.44
>50 years	10	82	
Ambulatory ERCP	10	81	0.51
Hospitalized ERCP	11	64	
Dilated bile duct by imaging studies pre-ERCP	14	104	0.58
Without dilation bile duct by imaging studies pre-ERCP	7	41	
Diameter of the bile duct by ERCP (mm)	9.0 ± 2.1	11.9 ± 5.0	0.001
With previous cholecystectomy	13	69	0.12
Without previous cholecystectomy	8	76	
Elevated pre-ERCP bilirubin	18	99	0.12
Normal pre-ERCP bilirubin	3	46	
Number of attempts to cannulate the biliary tract	9.1 ± 2.7	7.1 ± 3.5	0.02
Difficult cannulation			
<8 attempts	5	83	0.005
>8 attempts	16	62	
Precut (access) sphincterotomy			
Yes	17	78	0.01
No	4	67	
Biliary sphincterotomy			
Yes	11	86	0.54
No	10	59	
Cannulation time of the bile duct (min)	8.7 ± 2.8	6.1 ± 3.5	0.001
ERCP Length (min)	30.0 ± 3.7	23.7 ± 7.2	0.001
Pancreatography			
Yes	17	62	0.002
No	4	83	
Number of passes of the guide in the Wirsung duct.	2.3 ± 0.76	1.40 ± 0.75	0.000
Number of injections into the Wirsung duct.	2.0 ± 0.72	1.43 ± 0.64	0.001
Pancreatography extension			
Partial	2	9	0.39
Full	13	52	
Acinarization	2	1	
Serum amylase at 2 hours post-ERCP (U/L)	1163.5 ± 999.6	176.9 ± 105.2	0.001

**Table 5:** Summary of results of 11 published studies and the results of the present study

Author, year, country	Intervention	Inclusion criteria	Does the study include patients with a pancreatic stent for PEP prophylaxis?	Post-ERCP pancreatitis incidence				Risk Ratio, 95 % CI
				Experimental		Control		
				Events	n	Events	n	
Murray, 2003, Scotland. [14]	100 mg rectal Diclofenac in recovery area.	High risk patients	Yes	7	110	17	110	0.56 [0.29, 1.05]
Sotoudenhmanesh, 2007, India. [15]	100 mg rectal Indomethacin immediately prior to ERCP.	Unselected patients	No	7	221	15	221	0.62 [0.34, 1.16]
Montaño-Loza, 2007, Mexico. [23]	100 mg rectal Indomethacin, 2 h before ERCP.	Unselected patients	No	4	75	12	75	0.47 [0.20, 1.12]
Cheon, 2007, USA. [24]	50 mg Diclofenac, before and after ERCP by mouth	Unselected patients	Yes	17	105	17	102	0.98 [0.68, 1.42]
Khoshbaten, 2008, Iran. [22]	100 mg rectal Diclofenac on arrival to recovery area.	High risk patients	Yes	2	50	13	50	0.24 [0.06, 0.87]
Senol, 2009, Turkey. [25]	75 mg diclofenac IM and IV isotonic, after ERCP	Unselected patients	No	3	40	7	40	0.57 [0.21, 1.50]
Otsuka, 2012, Japan. [26]	50 mg rectal Diclofenac, 30 min before ERCP.	Unselected patients	No	2	51	10	53	0.31 [0.09, 1.12]
Elmunzer, 2012, USA. [16]	100 mg rectal Indomethacin after ERCP.	High risk patients	Yes	27	295	52	307	0.67 [0.49, 0.92]
Döbrönte, 2012, Hungary. [27]	100 mg rectal Indomethacin, 10 min before ERCP.	Unselected patients	No	11	130	11	98	0.87 [0.56, 1.34]
Abu-Safieh Yasser, 2014, Palestine. [28]	75 mg diclofenac IM, prior the ERCP.	Unselected patients	Yes	6	89	12	93	0.66 [0.34, 1.29]
Döbrönte, 2014, Hungary. [29]	100 mg rectal Indomethacin 10–15 min before ERCP.	Unselected patients	No	20	347	22	318	1.10 [0.82, 1.49]
Present Study, 2015, México.	100 mg rectal Indomethacin, immediately after ERCP.	High risk Patients	No	4	82	17	84	0.35 [0.14, 0.87]

Other complications included minor bleeding in 2 patients of the study group and 3 patients in the control group (*P* = 0.99). None of these patients required surgical treatment to resolve the complication. No perforations were observed. The only side effect observed was itching in the anus in 2 patients in each group. There was no mortality. The comparison between groups that developed and did not develop PEP is described in Table 4.

## Discussion

ERCP has become an essential therapeutic modality for pancreatic and biliary diseases since the introduction of endoscopic sphincterotomy [20]. Acute pancreatitis remains the most common complication of ERCP. Other complications such as hemorrhage, perforation, cholangitis and cholecystitis are observed with a lower incidence; however, these complications are no less important because they can lead to significant morbidity and mortality [17–19, 21].

The overall incidence of PEP in our study was 12.6 %, which is comparable to that reported in other series, considering that high-risk patients were studied. The frequency of PEP was higher in females (17 females versus 4 males), a finding that is also consistent with those of other prospective studies [14–18].

Our results showed that the use of indomethacin administered rectally compared with glycerin decreased the incidence of PEP in patients at a high risk of developing this complication (4.87 % versus 20.23 %), a difference that was significant (*P* = 0.01). The clinical and statistical significance of the intervention was expressed by an ARR of 0.15 (15 %), RRR of 0.75 (75 %) and a NNT of 6.5 patients to prevent one episode of pancreatitis.

Since 2003, 11 studies have been published regarding the preventive effect of NSAIDs in patients undergoing ERCP (Table 5). Murray et al. published the first randomized clinical trial [14]. They compared the use of 100 mg of rectally administered diclofenac versus placebo in the recovery area after ERCP, including 110 patients in each group. Risk factors for pancreatitis included as variables were pancreatography and SOD. The incidence of pancreatitis in the placebo group was 15.5 % (17/110) and 6.4 % (7/110) in the diclofenac group (*P* = 0.049).

In 2007, Sotoudehmanesh et al. compared the use of 100 mg of rectal indomethacin with placebo, administered immediately before the ERCP. They enrolled a heterogeneous group of 442 patients without distinguishing the presence of any high-risk group. The incidence of PEP in the placebo group was 6.8 % (15/221) and 3.2 % (7/221) in the indomethacin group, although this difference was not significant (*P* = 0.06). A post hoc subgroup analysis of patients undergoing pancreatography showed a significant protective effect of indomethacin (indomethacin 2.3 % versus placebo 18.6 %; *P* = 0.014) [15].

Moreover, in 2007, Khoshbaten et al. compared the use of 100 mg of rectally administered diclofenac compared with placebo, applied in the recovery area. They only included patients considered to be at high risk of PEP (patients undergoing pancreatography with or without cholangiogram). The incidence of PEP in the placebo group was 26 % (13/50) and 4 % (2/50) in the diclofenac group; this difference was significant (*P* ≤ 0.01) [22].

Montaño et al. conducted a study that involved 150 patients, comparing 100 mg of rectally administered indomethacin with placebo, applied before the procedure. In this study, patients undergoing ERCP for suspected biliary obstruction were included (they were not considered at high risk of PEP). They found an incidence of pancreatitis in the placebo group of 16 % (12/75) and 5.3 % (4/75) in the indomethacin group; this difference was significant (*P* = 0.034). All cases of pancreatitis were categorized as mild according to the consensus criteria, as occurred in our series [23].

In 2007, Cheon et al. published the results of a clinical trial in the USA, including patients at high (179/207) and low (28/207) risk of developing PEP. They compared the administration of 50 mg of oral diclofenac against placebo, applied 30 to 90 minutes before and 4 to 6 hours after ERCP. The incidence of pancreatitis in high-risk patients in the placebo group was 18 % (16/89) and 17.8 % (16/90) in the diclofenac group. The difference in the incidence and severity of pancreatitis between the two treatment groups was not significant [24].

More recently, Senol et al. assessed 80 patients and compared the administration of 75 mg of intramuscular diclofenac followed by infusion of 0.9 % NaCl for 4 hours (5–10 ml/kg) versus placebo (infusion of 500 cc of 0.9 % NaCl for 4 hours). The intervention was performed immediately after ERCP. The incidence of pancreatitis in the placebo group was 17.5 % (7/40) and 7.5 % (3/40) in the diclofenac group. This difference was not significant (*P* = 0.176), which may have been because of the small number of patients included [25].

In the same context, Otsuka et al. compared the administration of 50 mg transrectal diclofenac against placebo (glycerin suppository) in 104 patients, applied 30 minutes before ERCP. They reported an incidence of pancreatitis of 3.9 % (2/51) for the diclofenac group and 18.9 % (10/53) in the control group (*P* = 0.017) [26].

Elmunzer et al. conducted the most important controlled clinical trial, in which they enrolled 602 patients and compared 100 mg transrectal indomethacin against placebo (glycerin suppository) [15]. The intervention was performed immediately after ERCP. The incidence of pancreatitis in the placebo group was 16.9 % (52/307) and 9.2 % (27/295) in the indomethacin group, which was a significant difference (*P* = 0.03). It should be noted that in this study the authors placed a pancreatic stent in 246 patients in the indomethacin group (83.4 %) and 250 individuals in the placebo group (81.4 %). In addition, it should be highlighted that most of the patients were evaluated by clinical suspicion of SOD and over 15 % of cases and controls had a history of PEP.

Döbrönte et al. conducted a clinical trial that included 228 patients and evaluated the rectal application of 100 mg indomethacin against placebo (glycerin suppository), administered 10 minutes before performing ERCP. The incidence of pancreatitis in the indomethacin group was 8.4 % (11/130) and 11.2 % (11/98) for the control group, showing no significant difference (*P* = 0.48) [27]. This controlled clinical trial had an imbalance in the number of controls with a difference of 32 patients at the time of publishing, so the authors’ conclusions are poorly supported.

Abu-Safieh et al. conducted a randomized double-blind controlled trial in Palestine, including a total of 182 patients and comparing the intramuscular administration of 75 mg diclofenac with 3 ml of isotonic saline as a placebo. They reported an overall incidence of PEP of 10 %, 6.9 (6/89) for the diclofenac group and 12.9 % (12/93) for the placebo group. There was no significance difference in the incidence of PEP between the two groups (*P* = 0.164) [28].

Döbrönte et al. recently published the results of a multicenter clinical trial in 665 standard-risk patients divided into 347 study patients that received 100 mg of indomethacin before ERCP and 318 controls that received a placebo. The incidence of PEP was 5.8 % and 6.9 % (*P* = 0.54) [29]. As in their previous study [27], there was an imbalance in the number of controls with a difference of 29 patients at the time of publishing, so the authors’ conclusions may be insufficiently supported because of this difference.

From 2008 to the present, at least 10 meta-analyses have evaluated the results of the different clinical trials that have been reported. The results allow us to conclude that NSAIDs such as indomethacin or diclofenac used in the different routes of administration reduce the incidence of asymptomatic hyperamylasemia, pancreatitis and moderate to severe episodes of pancreatitis [12, 13, 30–37].

The results of our study are relevant because the drug was administrated immediately after completion of the endoscopic procedure, as was performed by Murray [14], Khoshbaten [22] and Elmunzer [16]. Only the study reported by Elmunzer used indomethacin; in the other two studies, diclofenac was preferred. The main difference between our study and that reported by Elmunzer was the combined use of pancreatic stenting in more than 80 % of patients in the latter. In our study, pancreatic stenting was only used to treat pancreatic fistulas.

Traditionally, it has been considered that the placement of a small caliber (5 Fr) stent in the pancreatic duct was the standard treatment to prevent this complication. It has also been recommended in the management guidelines for the prevention of pancreatitis in patients considered to be at high risk [5–7]. Recently, Akbar and colleagues published the results of a meta-analysis in which a total of 29 studies were included (22 with pancreatic stent placement and 7 with the use of NSAIDs), showing that stenting or transrectal administration of NSAIDs was superior to placebo in the prevention of PEP. The combination of transrectal application of NSAIDs and the use of stents showed no greater effectiveness in the prevention of PEP when compared with that of each intervention alone. The results further demonstrated that transrectally administered NSAIDs alone were superior to pancreatic stenting in preventing PEP (OR 0.48, 95 % CI, 0.26 to 0.87) and must be regarded as the first-line preventive therapy [32].

Recently, the United States Cooperative for Outcomes Research in Endoscopy group [38] published a post hoc analysis of the randomized controlled trial published by Elmunzer and colleagues [16]. They found that the incidence of PEP in placebo patients who received a failed pancreatic stent (FPS) was 34.7 %, and in those patients in this group with a successful pancreatic stent (PS), the incidence was 16.2 % and in patients without a PS it was only 12.1 %. In contrast, in patients who received a suppository of 100 mg of indomethacin immediately after the ERCP, the incidence of PEP was 5.3 % in the FPS patients, 9.6 % in patients with a successful PS and 10.3 % in those patients without a PS. This study reveals the important and relevant results of prophylactic use of rectal NSAIDs.

However, to support the previous conclusion, a high-quality multicenter randomized clinical trial is required to better understand the efficacy of pancreatic stents with and without rectal NSAIDs and with rectal NSAIDs alone to prevent PEP in high-risk patients.

Another important issue to consider is the cost of prophylactic treatments. In the hospital where this study was conducted, the cost of a suppository of 100 mg of indomethacin was USD $0.08, the glycerin suppository had a cost of USD $0.20 and the pancreatic endoprothesis had a cost of USD $350.00.

## Conclusions

This study showed that indomethacin administered rectally immediately after ERCP reduced the incidence of PEP in high-risk patients.

## Abbreviations

ERCP: Endoscopic retrograde cholangiopancreatography
PEP: Post-ERCP pancreatitis
SOD: Sphincter of Oddi dysfunction
NSAIDs: Nonsteroidal anti-inflammatory drugs
FPS: Failed pancreatic stent
PS: Successful pancreatic stent
